# Islam and the COVID-19 Pandemic: Between Religious Practice and Health Protection

**DOI:** 10.1007/s10943-021-01346-y

**Published:** 2021-07-15

**Authors:** Aldona Maria Piwko

**Affiliations:** Institute of Research in the Discipline of Political Science and Administration, Vistula University Warsaw, Stokłosy 3, 02-787 Warsaw, Poland

**Keywords:** Middle East, Religion, Policy, Islam, COVID-19 pandemic, Health protection

## Abstract

This paper concerns a problem, the global pandemic COVID-19, which has influenced religious practices with respect to health protection across the Muslim world. Rapid transmission of the virus between people has become a serious challenge and a threat to the health protection of many countries. The increase in the incidence of COVID-19 in the Muslim community took place during and after the pilgrimages to Iran's Qom and as a result of the Jamaat Tabligh movement meetings. However, restrictions on religious practices have become a platform for political discussions, especially among Muslim clergy. This paper is an analysis of the religious and political situation in Muslim countries, showing the use of Islam to achieve specific goals by the authorities, even at the price of the health and life of citizens.

## Introduction

Health in the Islamic tradition is closely related to the Muslim image of God and man. Thus, it is inseparable from sin, disease and suffering. Properly practicing the principles of religion provides the Muslim believer with inner balance and harmony in life. The importance of health in the concept of religion was emphasized in the holy book of Islam—Qur'an—which contains the principles of health and disease, while indicating the appropriate dietary and hygienic habits that ensure the health of the body. These recommendations are treated by believers as constitutive elements of Muslim health education, closely connected with religious practices. The word Islam in Arabic means submission to God, and therefore, all believers are bound to obey God's commands and prohibitions. Every Muslim must practice religion according to the Five Pillars of Islam, which are: Shahada—Profession of Faith; Salah—Prayer; Zakat—Almsgiving; Sawm—Fasting and Hajj—Pilgrimage (Lunde, 2002). Prayer is a guarantee of the health of the soul, because it calms the hearts of believers, and a healthy human soul contributes to the health of the whole organism. Almsgiving helps to free a person from the lust to possess and selfishly accumulate goods, only for himself. Helping the needy brings comfort to the believer and is a form of inner purification. Pilgrimage aims to free Muslims from everyday worries and troubles, and at the same time to make them aware of equality and belonging to the entire community of believers (Klöcker, 2002).

Islam is a monotheistic religion, the second largest religion in the world after Christianity. It is assumed that today this religion is professed by over 1 billion 926 million people worldwide (Zurlo, 2021). This study presents the results of research on the influence of the COVID-19 pandemic, attitudes of Muslim communities toward the risk of a disease with a high contagious factor and religious leaders' responses to WHO's recommendations in the context of religious practices. An important element of the article is to show the perception of the epidemic threat by Muslim leaders and using it in political activity. It should be noted that the opinion of Islamic religious leaders is very important to Muslims. Often the opinion of imams has greater moral authority than the appeals of politicians and doctors.

Religious requirements that must be met by Muslims can be (apart from pilgrimage) realized individually in their own homes. However, the century-old tradition of Islam has developed in the followers of Islam the belief that practicing religion together strengthens ties not only with God, but also with people, brothers in faith. For this reason, joint meetings in prayer are a particularly important element of religious life. Islam is often called the religion of prayer. In Qur'an, the words “Establish prayer and give zakāh” were repeated many times (The Qur’an, 2008). The honest fulfillment of this divine command is an expression of human thanksgiving to Allah for his life, as well as an act of praise to God and an expression of limitless submission to His will.

The fulfillment of these duties, however, changes with the onset of communicable diseases in the Islamic community. Prophet Muhammad himself recommended that during the plague, Muslims should limit all mobility: traveling, visiting each other and avoiding the sick. Muhammad also encouraged quarantine to keep the plagues from spreading. Sunnah clearly emphasizes: “If you hear that there is a plague in a land, do not enter it; and if it (plague) visits a land while you are therein, do not go out of it” (Sahih al-Bukhari, 2005b).

Thus, it can be concluded that Prophet Muhammad has already introduced strategies that are implemented in modern times by public health organizations such as the center for disease control. The rules for dealing with infectious diseases and, therefore, above all, the maintenance of cleanliness and hygiene, promoted by the Islamic health science, were much ahead of the times of Prophet Muhammad. Anyway, Islam very clearly emphasizes the importance of hygiene in human life. This is evidenced by the need to perform ritual ablutions before each ritual prayer. It should be emphasized here that Muslims are obliged to pray five times a day.

The COVID-19 (Gorbalenya, 2020; Chen, 2020) disease, caused by the Coronavirus SARS-CoV-2 (Cohen & Normile,^2020^), originally identified in November 2019 in Wuhan (Gao & Sai, 2021), China, and the latest research indicates it was already present in October 2019 in Hubei Province (Pekar et al., 2021), and started a global pandemic, changing the way of life of all people. Intensive transmission of the virus and numerous infections have forced the introduction of national and national restrictions aimed at limiting the mobility of the population, thus stopping the spread of the pandemic. There are suspicions that the virus originated in a laboratory, but there is no scientific evidence for it. WHO agreed to sponsor the first phase of an investigation into the pandemic’s origins, which took place in China in early 2021 (Maxmen & Mallapaty, 2021). Dr Francis Boyle believes the virus is potentially lethal and an offensive biological warfare weapon or dual-use biowarfare weapon agent genetically modified with the gain of function properties (Skopec, 2021). This thesis requires in-depth research, but in the face of the unpredictability of the terrorist activities of jihadists of the Islamic State, it should be borne in mind (Bandeira et al., 2021). SARS virus stands for severe acute respiratory syndrome coronavirus 2 and is the third virus in the last 20 years to cause a worldwide epidemic. The typical symptoms of the disease are fever, dry cough, fatigue and shallow breathing. Less common are: sore throat, runny nose and sneezing. Most of those infected have mild symptoms and the disease itself is mild (Hui, 2020). However, these people can infect others. COVID-19 disease can be very severe, leading to pneumonia and multiple organ failure and death. Severe course of the disease requires mechanical respiratory ventilation. The elderly and chronically ill are particularly vulnerable to this course of disease. The incubation time of the disease ranges from 1 to 14 days, but most often the first symptoms appear 5–6 days after infection. The virus is spread by airborne droplets (Chen, 2020). The intensity of transmission of the virus outside China and numerous deaths caused the World Health Organization to recognize the infectious COVID-19 disease caused by the coronavirus as a pandemic on March 11, 2020. By the end of June 2021, 183 million people had become infected worldwide and almost 4 million people had died from COVID-19 (WHO, 2021a).

The pandemic has affected every area of life: social, economic, economic and religious (Androutsopoulos, 2021; Sułkowski & Ignatowski, 2020). In addition, almost all human life has been transferred to virtual space (Mishra et al., 2020). Numerous sanitary restrictions aimed at preventing the spread of the virus made it impossible to gather for community prayer in churches, synagogues, mosques and other places of prayer. Restrictions on gathering people to meet religious needs have been in place in many places around the world (Boguszewski et al., 2020; Begović, 2020; Al-Astewani, 2021; Osei-Tutu et al., 2021; Kostecki & Piwko, 2021; Kühle & Larsen, 2021). For this reason, almost all religious activities have been transferred to television and the Internet (Molteni et al., 2020). Religious rituals and practices were carried out in virtual reality. Thus, there was an intensive mediatization of religiosity (Tudor et al., 2021; Przywara et al., 2021). The COVID-19 pandemic also contributed to a deeper search for faith and spirituality (Bentzen, 2020). Compliance with sanitary recommendations in the religious sphere was implemented in various ways. Some believers followed the restrictions without questioning their validity. Others saw the restrictions as an act of evil force aimed at destroying religiosity (Wildman et al., 2020).

In the face of COVID-19 pandemics, the Islamic community was faced with the problem of reconciling the high religious requirements for God that believers should meet and ensuring the safety of their own health and ummah. During this difficult time, religious leaders can play a major role in saving lives and reducing illness related to COVID-19. They are a primary source of support, comfort, guidance and direct healthcare and social service, for the communities they serve. The message of the religious leaders was more acceptable than warnings from other sources (WHO, 2020a).

The main research goal of the article is to analyze the correlation between practicing the religion of Islam and health protection in the context of sanitary safety and health policy in Muslim countries during the COVID-19 pandemic. For this reason, the presented topic is interdisciplinary, as it combines issues typical of religious studies, Islamic studies and political science research on health protection. The main questions posed in the article are: Are religious practices a carrier of an epidemic threat? Can sacred rites be dangerous and lead to the death of their followers? Are religious and political leaders in the Muslim world acting to the detriment of citizens by spreading unconfirmed reports on the COVID-19 pandemic? Is the reaction of the Islamic clergy to the need to close mosques? An important question in the context of the current pandemic is also the question of the health of Muslim refugees staying in camps. While the issues related to the sanitary and humanitarian quality of refugee camps have been discussed many times in the literature, they have never been analyzed in the context of a global pandemic (Robinson, 2020).

## Materials and Methods

Muslim humanitarian organization Islamic Relief, guided by the motto: Islamic law, like humanitarian work, is based on the broader principle of ‘do no harm’, provided guidelines on safe religious practice during the coronavirus pandemic. Atallah Fitzgibbon Faith Partnership Advisor at Islamic Relief Worldwide, he stressed that religious practices should be adjusted, including closing mosques for believers, in order to ensure health safety for the public. These principles are justified by Islamic law and medical knowledge about COVID-19 (Islamic Relief, 2020).

The article is a response to current events in the Islamic world related to the coronavirus pandemic. The COVID-19 pandemic has become an element of political activity, especially in countries with regimes. Iran is a clear example of using a pandemic for its own purposes (Blandenier, 2020). Also the leaders of the so-called Islamic State saw the possibility of extending their influence thanks to the pandemic, presenting it as God's punishment on the infidels (Daymon & Criezis, 2020).

The analysis covers the Iran, being the center of shiism, in which clerics play an important role in shaping public opinion. The choice of Iran is also important due to the country's political situation and the numerous international restrictions imposed on it (Dubowitz & Ghasseminejad, 2020). Religious groups were also analyzed: Jaamat from East Asia (Burhani, 2020; Bustamam-Ahmad, 2008) and ISIS (Krona & Pennington, 2019; Nacos, 2016). The decisive factor in the selection of countries and groups to be analyzed was their importance in the Muslim world and thus their impact on a significant number of people and political importance in the international arena.

Detailed data on the number of coronavirus infections were obtained from databases of the World Health Organization and official government communications of each country (WHO, 2021a). These data are constantly updated due to the dynamic development of COVID-19 pandemics. The collected material was ordered and systematized. The study used the comparative method of Max Weber (Weber, 1949), which consists in describing the phenomena related to the perception and commenting on the spread of COVID-19 pandemics in the analyzed Muslim societies. The comparative method also made it possible to explain the similarities and differences between the same social phenomena, which is COVID-19 pandemic, occurring in different Islamic societies at the same time. And the main research technique is qualitative content analysis. The fundamental element of the study was to define the perception of health in the context of religious requirements arising from Islam. Descriptive and analytical methods were used to present the results of the study, which showed how religious authority influences the community of believers.

The study used in-depth interviews with Muslims from Iran, Egypt and Turkey, as well as with representatives of the Shi'ite clergy. Fifteen people participated in the study: 8 men, including 3 Shi'ite clergymen and 7 women. In this group, 8 people were representatives of Shi'ite Islam, and 7 people professed Sunni Islam. Six people were from Iran, 6 from Turkey and 3 from Egypt. Due to the pandemic and the limited possibilities of field research, electronic means of communication and the Internet were used for the interviews. The interviews were conducted via the social network Facebook as well as via e-mail correspondence. The criteria for selecting people for the study were: country of residence, active religion of Shi'ite or Sunni Islam, at least secondary education, communicative knowledge of the English language. People known to the researcher who also knew the researcher participated in the study. The respondents knew what the purpose of the survey was for. Therefore, it cannot be concluded that the selection of the sample was accidental and the respondents constitute a representative research group. On the other hand, all people knew the situation in their country and experienced difficulties with religious practice, and some of the respondents participated in the pilgrimage to Qom. The study was conducted using a structured interview. The questions were developed in advance and were asked to respondents in the same order. The research was conducted in 2020. However, this issue is still relevant because the development of the pandemic is dynamic. More waves of infection are coming and they are affecting the religious life of all societies in the world.

## Results

The Middle East is a sensitive region, not only because of the armed conflicts there and the conflicting business, trade and economic interests of many countries. The migration crisis in Europe, initiated in the Arab spring and then the outbreak of the civil war in Syria, which peaked in 2015, is described as one of the largest that Europe has faced since the end of World War II. Movement of the population was closely related to the transmission of various diseases: mild and typical for numerous human clusters, but also dangerous, including infectious ones, which were combated in Europe thanks to the operation of preventive programs. World Health Organization, Regional Office for Europe in Report on the health of refugees and migrants in the WHO European Region (WHO, 2018). No public health without refugee and migrant health argued that refugees and migrants did not bring Europeans an increase in infectious diseases. Experts emphasized, however, that the Western lifestyle is dangerous to the health of newcomers from other regions of the world. While it is true that migrants bring disease with them, the risk of it spreading to the indigenous peoples of Europe is negligible. Research has shown that migrants, upon arrival in Europe, are less likely to suffer from non-communicable diseases, such as cancer, heart attack, stroke, diabetes and obesity. However, after some time in highly developed countries, usually after five years, they begin to suffer from civilization diseases: obesity, hypertension and heart disease, as often as European communities (WHO, 2018).

The sociopolitical instability of the Middle East in the face of the coronavirus pandemic is currently a particular threat to the sanitary safety of not only the population of the region, but also other parts of the world. The ongoing conflicts in Yemen and Syria make it almost impossible to detect the infected and monitor the spread of the virus. An additional obstacle in counteracting the epidemic is the ineffective healthcare system and inadequate securing of national borders. In four countries: Iraq, Syria, Lebanon and Turkey, 12 million people are internally displaced people who pose a particular epidemiological threat. Inappropriate living conditions, especially in overcrowded refugee camps, where there is limited access to sanitary facilities with running and hot water, as well as the lack of basic hygiene measures, as well as the lack of proper medical care, increase the risk of contracting COVID-19.

For this reason, the COVID-19 pandemic is particularly dangerous for people in refugee camps, especially those heavily overcrowded in Iran, Afghanistan, Bangladesh and Greece. On the Greek island of Lesvos, there is the largest refugee camp in Europe, called Moria, which was prepared for three thousand people. Currently, there are 26,000 people there. The administration introduced a ban on leaving the camp from 7 pm to 7 am. This restriction was introduced to minimize the risk of coronavirus infection. In 2020, the Dutch Government donated medical equipment to Mavrovouni's medical hub in Moria Refugee Camp. To prevent and mitigate the spread of the pandemic, asylum seekers have undergone rapid COVID-19 testing. People with a positive result are referred to isolation cells. There are currently no active COVID-19 cases in Moria (UNHCR, 2021a).

A similar situation occurs in the Zaatari Refugee Camp in Jordan, where 80,000 Syrian displaced persons are held. Improper sanitary standards are the biggest problem in the camp, and these include: unsystematic garbage collection, non-working air conditioning, difficulties with access to bathrooms and lack of running water. These problems were present in the pre-COVID-19 pandemic camp and resulted in diseases such as scabies and lice. In order to reduce the number of coronavirus infections, information campaigns are conducted in the camp to promote a hygienic lifestyle. Access to clean water has been increased from 35 L per person to 60 L per person per day (UNHCR, 2021b).

A threat to sanitary safety is also posed by the armed conflicts in the Middle East countries. Both Syria and Yemen are in deep chaos. The Syrian civil war collapsed the country's healthcare system and sanitary infrastructure. In addition, the country is still politically correct, in which the government claims that there are no coronavirus infections, and medical personnel are prohibited from reporting cases of infections. However, the WHO data show that over 24,000 people have been infected (WHO, 2021b). However, it should be emphasized that the country is not able to control the health of its citizens due to the practically nonexistent infrastructure. The International Rescue Committee has issued a warning that the outbreak of the pandemic in northern Syria could be one of the worst in the world (UN, 2020). At the same time, it should be emphasized that as a result of the hostilities in the Idlib Province, nearly a million people had to leave their homes and headed toward Turkey. At the same time, Turkey opened its border with Greece for refugees. Such activities contribute not only to the further transmission of infections, but most of all they pose a significant threat to the health and life of the refugees themselves.

In Yemen, an armed conflict with Saudi Arabia has been ongoing since 2015. The Yemeni rebels of the Houthi tribe are supported by Iran, which is currently the epicenter of coronavirus infections in the Middle East. For this reason, the UN Envoy for Yemen, Martin Griffiths, and the UN Secretary General, Antonio Guterres, called for an end to military activities in the region in view of the pandemic. The World Health Organization, on March 26, 2020, announced that there is no confirmed case of coronavirus infection in Yemen to date (WHO, 2020b). However, now in a country covered by a civil war, almost 7000 cases of the disease have been confirmed. The lack of sufficient medical care and the limited possibilities of counteracting the pandemic cause tragic consequences. Millions of people do not have access to clean water and hygiene products.

### Restrictions on Muslim Religious Practices

In connection with the COVID-19 pandemic, state authorities have implemented numerous restrictions on civil liberties aimed at stopping the spread of the infection. Sanitary restrictions have also been applied in religious practices.

Coronavirus affects not only the physical part of people, causing serious illness and even death. The high transmission of the virus between people forced spiritual leaders to change the organization of religious practices. Every follower of Islam, always before praying, must perform ablution, consisting in ritual washing of the body. Cleaning should be done with water, but in the absence of water, it is acceptable to use a clean felt-tip pen or gravel, e.g., when traveling. That is why there are bathhouses in mosques and houses of prayer, in which ritual washing should be performed. Despite such an obligation, mosques have introduced a ban on gathering for common prayer. A Shi’ite man from Iran: “I know that you can get infected with the virus, but I want to pray in a mosque, listen to Jumaa Khutbah, and we were forbidden to do so, I do not understand this decision.” Turkish, sunni woman: “according to Islam, prayer contributes to the health of the soul, and since the soul is healthy, the body will also be. Prayer in a mosque should not be forbidden. Obviously, it is necessary to maintain hygiene and social distance.” Additionally, it was forbidden to bring your own prayer rug to the mosque. Man from Egypt: “The world has turned upside down. Everyone is afraid of disease and they have forgotten God. Even your own sajjada rug is forbidden in the mosque because it is supposed to be infected.” Fearing an intense increase in infections, Saudi Arabia closed Mecca to pilgrims, the holiest city in Islam, where the only Muslim temple is located. Every believer should at least once in his life make a ritual pilgrimage to Mecca, at the time prescribed by religion. In 2020, the time of the most important religious event was from July 29 to August 3 (Lunde, 2002).

On the other hand, at any time, a believer can make a little pilgrimage called the umrah. This possibility is used by many millions of Muslims every year. In Mecca and in Medina, Islam's second holy city, where the tomb of Muhammad is located, Muslims flock throughout the year. Therefore, on February 27, 2020, the Saudi authorities decided to close the country's borders to all those traveling to the country for religious purposes. The restrictions introduced by the Saudi Arabian authorities aroused concern among the followers of Islam, because from April 24 to May 23, 2020, the month of Ramadan fell on the Muslim calendar, in which Muslims lived the holy time of fasting and often traveled to Mecca and Medina as part of in-depth prayer. Egyptian man: “I am disappointed with the decision of the Saudi Arabian authorities not to make a pilgrimage to Mecca in 2020. I really wanted to make a pilgrimage because it is an important year in my life. I turned 50, my grandson was born, I wanted to fulfill my duty.” Similar solutions regarding the limitation or complete closure of pilgrimage sites were made by the Iraqi authorities, suspending pilgrimages to shrines in Najaf and Karbala, which are holy places for the Shi'ite community. In Morocco, too, the annual moussems celebrated in honor of saints or marabou have been canceled. The local, often rural tradition is deeply rooted in the mentality of the Moroccans, and therefore, it is an essential part of the community life of the Muslim community. The COVID-19 pandemic has forced believers to change religious practices (Hoang, 2020; Atique & Itumalla, 2020). Imam from Turkey: “We have to apply a sanitary regime, so the mosque is closed. Anyone can pray at home. I am also always ready to help those in need, you can always talk to me in person and by phone. I do not want to undermine the decisions of state authorities.”

In 2021, Saudi Arabia eased restrictions related to COVID-19 pandemics during the month of Ramadan and Eid al-Fitr holidays. However, precautionary measures are still recommended, such as social distancing and hand disinfection. Other Muslim countries, such as Egypt, Tunisia and Iraq, have restricted their shops during Eid al-Fitr holidays to avoid crowds in shopping malls (Al-Jazeera, 2021). A young woman from Egypt: “They talk constantly on TV, not to gather for prayer in the mosque, but you can go to the shopping center and the bazaar. So you can spend money, there is no virus threat, but not to meet friends on Eid.”

### COVID-19 Pandemic in Iran

The epicenter of the COVID-19 pandemic in Iran is the city of Qom with over a million inhabitants, the holy Shi'ite city and their pilgrimage destination (Seyfi, 2018). It is estimated that more than 20 million believers come to the city annually to pray at the Fatima Masumeh mosque, the sister of the eighth imam in the Imamite tradition, and thus the mainstream Shi'ite. In addition, roads leading to 17 provinces of Iran intersect in Qom (Al-Rousan & Al-Najjar, 2020). The significant movement of people, coupled with a high concentration of religious practitioners, contributed to the spread of the infection in Iran rapidly. The first infection in Iran probably occurred on February 19, 2020. The virus was carried by a Chinese merchant from Wuhan who traded with Qom. The Shycikh clerical regime downplayed the threat, claiming that the isolation and quarantine of entire cities are an outdated and ineffective action. The head of the sanctuary in Qom, Mohammad Saeedi, called on pilgrims to make pilgrimages, claiming that the holy sanctuary was a place of healing and that coming to it would cure not only spiritual but also physical diseases (Fouladiyan, 2021). A Shi’ite woman from Iran: “I don't watch TV, but they scare people with diseases. There were always diseases. Qur'an is our health, you have to believe. This is what our imam says, and I believe him.”

Carelessness contributed to the escalation of the disease. The pilgrims infected in Qom are not only Iranians, but also people from Kuwait, Iraq, Lebanon, Bahrain and Afghanistan. Also, public health authorities in Qatar, Jordan, Egypt, Oman, Saudi Arabia and the United Arab Emirates reported that the virus was mainly infected in people returning from Iran (Wright, 2020). Iranian woman: “my cousin was on a pilgrimage to Qom, he came back healthy, he did not infect anyone. It seems to me that these are false accusations that the people of Qom got infected and carried the plague to other countries.”

Another aspect could have contributed to the large-scale transmission of the coronavirus in Iran. In many Asian countries, missionary activities are carried out by Iranian religious organizations whose goal is to convert Sunni communities to Shi'ite Islam. The Shi'ite faith is most often from Qom. Their mission is to encourage candidates to come to Iran, and the motivational element is often a high scholarship. China is a special place of vigorous missionary activity, only because of the persecution that Muslims have to deal within the country of the center. At the same time, this religious activity partially explains the reasons for the spread of the virus in Iran so rapidly (Maçães, 2020).

The intense increase in coronavirus infections in Iran is also the result of the policy pursued by the local regime. China is an important economic and strategic partner for Iran. Relevant agreements concluded between states on cooperation in the field of civil nuclear energy, reviving the new Silk Road as an element of trade, as well as close cooperation in the fight against terrorism and extremism in Iraq, Syria, Afghanistan and Yemen, are an element of mutual interest of states (Shariatinia, 2011). Probably for this reason, the authorities were reluctant to inform about the intensification of infections with a virus originating in China. Undoubtedly, this attitude of the state administration is influenced by the economic isolation of Iran and sanctions imposed on the country, for example by the USA. Therefore, the government seems to be worried about Iran's weakened economy more than it is worried about the deteriorating health of its citizens (Rabbani, 2021). The improper actions of the government in Tehran made Iran the next, after China, large center of the virus epidemic from which it spread in the region. The above actions of the authorities exposed citizens to the risk of losing their health and even life.

The Iranian authorities have tried to use the pandemic to have the USA lift the economic sanctions imposed on the country that make it difficult to fight the epidemic. The actions of the Americans were defined as medical terrorism because they prevented the purchase of hygiene products, medicines and food. Iranian demands were supported by the UN Secretary General Antonio Guterres, as well as China. However, the Washington administration remained firm, further increasing the scope of trade restrictions (Macias, 2020). Iranian Man: “This whole pandemic is against Iran. The Americans want my country to surrender.”

Thoughtful political actions were also attempts to portray the coronavirus pandemic as aggression by the USA against poor or economically weakened countries, including Iran. The Supreme Leader of the Islamic Republic of Iran, clergyman and politician Ali Chamanei, stated that the USA invented a special version of the COVID-19 disease virus in order to destroy his country. The clergyman believed that this was supposed to be a retaliation for Iran's nuclear plans. Ali Chamanei thus upheld the claims made by Chinese politicians. These statements clearly show the growing political tensions between the USA and Iran, and between the USA and China. Sunni woman from Egypt: “You are a Christian, and you don't know that Jesus healed? You have to believe and pray. Illness cannot distance man from God.”

### Religious Meetings of the Jamaat Tabligh Movement

The Indian-based Jamaat Tabligh Muslim social movement is a non-political association spreading the faith of the First Umma, encouraging Muslims to practice the religion of Muhammad during the lifetime of Muhammad. It focuses primarily on matters related to religious rituals, clothing and personal behavior (Timol, 2019). The movement is especially popular in South Asia, but it is also present in other parts of the world. Jamaat Tabligh is described as the most influential religious movement in Islam in the twentieth century. The movement restricts its activities to Muslims and does not confront other religious communities, nor did it show any political ambitions. It proclaims that politics is global, and thus distracts man from faith and love for God (Ahmad, 2021; Lone, 2018).

The movement held a massive meeting at the Sri Prtaling mosque near Kuala Lumpur, Malaysia's capital city, from February 27 to March 1, 2020, which proved to be the source of a significant number of coronavirus infections. The meeting was attended by 16,000 people, including over 1500 foreigners from several countries in the region: Indonesia, Philippines, Singapore, Brunei, Cambodia, Thailand and Vietnam, who transferred the infection to their countries. More than a dozen days after the meeting, Malaysian authorities said more than 600 infections out of 1000 cases of the virus had their source in the Jamaat Tabligh meeting. Despite all efforts, not all participants of the meeting were found and examined (Sarkar, 2020).

A similar four-day meeting of the movement was scheduled on the island of Celebes in Indonesia, the largest Muslim state in the world. However, the country's authorities suspended a mass gathering, and 9000 pilgrims who attended the meeting were quarantined to prevent transmission of the virus. The organizer of the event, Sentot Abu Thoriq, was disappointed with the government's decision to liquidate the event, which had been planned over a year earlier. He stated that health, sickness and death are God's destiny, and members of the movement believe that God will bless and protect the godly. Another organizing committee member said he feared God more than the virus. Egyptian woman: “Politicians only issue new bans, no one wondered why the plague came.” The words of the organizers fit in with the narrative adopted by some Muslim radical religious leaders. Earlier, one of the Iranian imams spoke similarly about the coronavirus pandemic (Gomez, 2020).

### ISIS Recommendations on COVID-19 Pandemic

Leaders of the so-called Islamic State, at the beginning of the pandemic, informed their supporters in the Al-Naba newsletter that the coronavirus is a punishment for communist China for the persecution of the Muslim Uighur minority (Rayila, 2011). Subsequently, the Chinese government was widely criticized for concealing the true number of those who died and who died from the disease. With the spread of the global COVID-19 pandemic, anxiety grew among the so-called Islamic State. In the early stages of the pandemic, ISIS leaders feared an infection spread by Chinese Uighurs recruited into the ranks of the organization. Probably for this reason, appropriate recommendations were issued for soldiers, known as Sharia directives, which were published in the ISIS bulletin “Al-Naba.” The foundation is the duty to trust God and seek refuge in Him, also from all diseases (Sunnah an-Nasa’i, 2009). However, further recommendations addressed to agents of the so-called Islamic State, concern bypassing Europe, called the land of epidemics in the manual. The comment in the ISIS bulletin on the plague, from the hadith collection, states that it is a God-sent torment, because diseases do not strike by themselves, but under the command of the Creator himself. Allah will make it an instrument of mercy for believers, patiently overcoming the scourge must have confidence in God. Man will meet what the Creator has prepared for him, it can also be a martyr's reward (Sahih al-Bukhari, 2005a).

The travel safety rules put in place by ISIS leaders are interesting as, until the COVID-19 pandemic outbreaks, militants were encouraged to plan attacks in Europe and travel to European countries to recruit new combat-ready troops. The rules for preventing transmission of the virus recommend that people who may have contact with the infected should not leave the region in which they are located. Administration of the so-called Islamic State is concerned about the spread of disease in its territory and among its soldiers. The directives also cover healthy people who should not enter pandemic areas (Azman, 2021). Members of a terrorist organization are also bound by strict rules of personal hygiene. The instruction requires you to cover your mouth when sneezing and yawning, and to wash your hands frequently. The obligation to maintain hygiene was supported by an appropriate hadith referring to germs and all contamination, in which the followers of Islam are instructed to cover the vessels and tie water bags, because it is night in the year when the plagues descend, they pass through the vessels that are not covered and some this blight may remain in the vessel or water bag (Sahih Muslim, 2009). On this basis, ISIS leaders have urged people to wash their hands regularly, especially before dipping them in any utensils (Johnson, 2020).

In addition to the instructions on the rules of sanitary safety, applicable to all members and supporters of the so-called Islamic State, an extensive article was published in the official Al-Naba newsletter, discussing the pandemic in the world. The authors point out that the COVID-19 pandemic, which mainly affected idolatrous nations, is an example of God's action, sending torments to the unbelievers and ensuring the safety of believers (Al-Naba, 2020b).

In the article, Christian communities, mainly European, were called crusaders. The actions of the leaders of countries affected by the pandemic in counteracting economic effects, including price instability, were also analyzed. The topic of internal security and the activation of the army in individual countries struggling with the pandemic were also discussed. An important element of the article was to emphasize the weaknesses of European countries as a result of the pandemic, while pointing to the concerns of European Union countries related to the imminent danger of terrorist attacks, similar to those that took place in Paris, London and Brussels. The article was also a form of empowerment and support for all militants, clearly indicating that despite the epidemic, they cannot abandon their path, which is jihad. Jihadist soldiers have been called to continue working to free Muslim prisoners from camps where they experience not only enslavement but also disease and harm from infidels. In addition, the weaknesses of European countries which, as a result of the spreading pandemic, had to close state institutions, as well as to cease economic activity on a large scale, were also indicated. All these activities made the authors of the article extremely happy, pointing to a kind of God's punishment for the harm done to Muslims (Jawad Al-Tamimi, 2020). Man from Turkey: “Of course I'm scared of getting sick, it's terrifying what they show on TV, but I'm much more afraid that ISIS will send out its fighters to infect other people.”

The so-called Islamic State has suffered a series of defeats in recent months, losing significant territory previously occupied. However, it is still active in parts of Iraq and Syria. The COVID-19 pandemic has also affected Middle East countries. ISIS also has its cells in Africa. However, so far a small number of infected have been recorded on the continent. However, this does not mean that the virus will bypass Africa. There is a justified risk of spreading the virus in an uncontrolled manner, associated with insufficient medical care and poor quality of its services, as well as low health awareness of the population, especially in rural communities and distant from large urban centers. Therefore, the Sharia directives also apply to centers of fighters, the so-called Islamic State in West and Central Africa. Due to the ongoing conflicts in Syria and Yemen, it is difficult to know at what rate and in what area the coronavirus is spreading. Thus, it is extremely difficult to assess the consequences of the COVID-19 pandemic in these areas. The concerns about the sanitary safety of ISIS fighters are therefore justified. Lack of regular control over internal migrations of people in conflict-affected areas may, to a large extent, result in unrestricted transmission of the virus (Al-Naba, 2020a).

## Discussion

The conducted research made it possible to answer the above questions. First of all, religious practices carried out in large groups of people, without maintaining an appropriate social distance and proper personal hygiene, may contribute to the intensification of the transmission of pathogenic viruses, especially those transmitted by airborne droplets. Examples of such transmission of COVID-19 were the pilgrimage to Iran's Qom and the religious meetings of the Jamaat Tabligh movement. However, it cannot be unequivocally stated that community religious practices are becoming a special plane contributing to the spread of diseases. Restricting community religious practices should be primarily aimed at educating on the prevention of the transmission of infectious diseases, and not completely closing access to the place of worship. It has been shown above that carrying out sacred religious rites during a global pandemic, such as pilgrimage in Islam, may contribute to an increase in the number of deaths among participants of these rites. However, the direct cause of the pilgrims' deaths was an infectious disease, and not the mere fact of participating in the pilgrimage and religious ceremonies. Failure to maintain an appropriate social distance and proper hand hygiene contributes to the uncontrolled transmission of the virus. The conducted research also showed a significant influence of the opinion of the Muslim clergy on the society. Opinions expressed by Shi'ite clergy that there was no possibility of contracting the COVID-19 disease during the prayer was not only inconsistent with the state of scientific knowledge, but above all extremely harmful to the population. It misled the population and thus could contribute to an increase in the number of cases. Finally, the decision to close mosques to the public was particularly hard to accept, not only for the clergy but also for the believers. All religious communities around the world had to face this problem. Restricting the use of places of worship has made believers of all religions feel abandoned by their religious leaders and alone in the face of the COVID-19 pandemic. These limitations showed people how important religion is in life, especially when health and life are at risk (Al-Astewani, 2021).

## Conclusions

The COVID-19 pandemic came as a surprise to the whole world. Although the population has got used to the threat to security, it is understood as a threat to peace, caused by terrorist attacks or armed conflicts.

The problem of threats to public health will continue to grow. Until a few years ago, such an intense escalation of a virus, causing an infectious disease, against which modern and modern medicine is almost helpless, was not imagined. There were concerns about refugees and forced migrants from regions with low sanitary status and inefficient medical systems. Meanwhile, the usual mobility of modern society has contributed to the extremely rapid transmission of COVID-19 disease around the world. The problem of the threat to health safety spreading in societies will increase in the future, due to significant disparities in health protection in different parts of the world; moreover, the mobility of the population is greater than ever before, and it is obvious that this will continue to increase.

Based on the study, it should be stated that the approach of the Muslim community to COVID-19 pandemics depends on three factors. The first is the religious commitment of the respondents. The study showed that the greater the religiosity and strong adherence to the religious practices of Islam, the lower the acceptance of pandemic restrictions. The second important factor is the distrust toward the decisions of politicians who interfered too intensively in religious life. People taking part in the survey indicated that the clergy was too docile to politicians. The imams tried not to question the validity of the restrictions introduced. Shi'ite clerics viewed the COVID-19 pandemic somewhat differently. Some of them questioned government and medical decisions in public, official statements. The third factor is the subjective assessment of world events.

The intense transmission of the virus, taking its death toll among humans, was simply unimaginable. It is the scale of the infection's range and the lack of experience in combating the disease pandemic in the modern world, on the one hand, contributed to the carelessness of people and often to disregard for the threat. It also contributed to the feeling of being lost and helpless in the face of the destructive force of nature. It is probably for this reason that some spiritual leaders of the Muslim world have tried with all their might to emphasize the importance and power of prayer during the COVID-19 pandemics. Of course, trust in God is an extremely important element to help us survive the difficult times, but it was a great mistake to oppose sanitary rules. Iranian imams did not make a timely decision to sanitary close down places of worship for pilgrims, especially in Qom. This decision significantly contributed to the intensification of the virus transmission not only in Iran but also in other countries. A similar disregard was demonstrated by the organizers of the meetings of the Jamaat Tabligh religious movement in Malaysia. The arguments raised by Muslim spiritual leaders regarding total trust in God during the epidemic were skewed and applied politically. This message had one purpose: to show the world beyond the Muslims that a terrible plague has struck the unbelievers. Unfortunately, the delayed implementation of sanitary safety rules in Muslim countries had a different effect. Iran has become the second largest coronavirus transmission epicenter after China. However, it is not known how many people were infected in their countries by people returning from Iran.

The range of infections with an infectious disease, unprecedented in recent decades, has shown deficiencies and insufficient procedures in the preparation of individual countries to counteract epidemics. The spreading coronavirus has also highlighted various health safety concerns. Among these is the question of refugee camps. The COVID-19 pandemic has also strengthened the position of individual European Union countries on the increased migration of people, mainly from the Middle East and North Africa to Europe. Closing borders for fear of transmission of the virus makes it much more difficult to apply for asylum. An additional obstacle is the non-functioning offices in many European Union countries, as well as the suspended implementation of aid programs for refugees.

The leaders of the Muslim world have tried to use the prevailing COVID-19 pandemic to fortify their own political and religious positions, often disregarding global sanitary standards, thus exposing citizens to the risk of contracting coronavirus that is difficult to treat. The pandemic also revealed the weakness of the sanitary and epidemiological facilities of many countries. In the case of Iran, a problem in properly managing the COVID-19 pandemic has been the economic constraints caused by the political sanctions Iran is facing. Improper access to drugs and medical equipment had a significant impact on the increase in the number of infected and dead people. There is no doubt, however, that ending the pandemic will require the implementation of new systems for preventing and combating infectious diseases.
